# Evaluation of mild hypothermia therapy for neonatal hypoxic-ischaemic encephalopathy on brain energy metabolism using ^18^F-fluorodeoxyglucose positron emission computed tomography

**DOI:** 10.3892/etm.2014.1884

**Published:** 2014-08-06

**Authors:** MEI LUO, QINGPING LI, WENBIN DONG, XUESONG ZHAI, LAN KANG

**Affiliations:** Neonatal Department, The Affiliated Hospital of Luzhou Medical College, Luzhou, Sichuan 646000, P.R. China

**Keywords:** ^18^F-fluorodeoxyglucose positron emission tomography computed tomography, mild hypothermia therapy, new-borns, hypoxic-ischaemic encephalopathy, glucose metabolism

## Abstract

It remains unclear whether mild hypothermia affects energy metabolism in the brain tissue of newborns with hypoxic-ischaemic encephalopathy (HIE). The current study aimed to investigate the effect of mild hypothermia on energy metabolism in neonatal HIE and assess brain energy metabolism using position emission tomography/computed tomography (PET/CT) scanning. The mean standardised uptake values of ^18^F-fluorodeoxyglucose (^18^F-FDG) were used to determine the glucose metabolic rate in various brain anatomical regions, including the thalamus, basal ganglia and the frontal, parietal and occipital lobes. The rate of glucose metabolism significantly improved following treatment with mild hypothermia therapy and conventional therapy (P<0.001). Prior to the treatment, no significant differences were identified between the groups (P>0.05). Following treatment, the rate of glucose metabolism was significantly improved in the mild hypothermia therapy group compared with that in the conventional therapy group (P<0.001). Thus, these results indicate that mild hypothermia therapy effectively promotes the recovery of patients with neonatal HIE. ^18^F-FDG PET/CT scanning may be used to provide reference values for the assessment of energetic metabolism in patients with neonatal HIE.

## Introduction

Hypoxic-ischaemic encephalopathy (HIE) is an anoxia- and ischaemia-associated secondary cerebral injury caused by various factors. It is a significant complication of neonatal asphyxia. HIE is also a leading cause of neonatal fatality and disability in children; therefore, it remains the focus for investigation in numerous studies (1). The pathogenesis of HIE remains unclear although, based on related studies, it has been considered as a multi-mechanism combination of anoxia- and ischaemia-induced energy exhaustion, reperfusion and oxidative stress, the toxic effects of excitatory amino acids (EAAs), inflammatory injury (2) and cell apoptosis (3). The morbidity rate of HIE is generally accepted to be between 2 and 9% (4,5). In addition to the classic symptomatic and supportive therapies, mild hypothermia therapy is the only specific neuroprotective procedure recommended for the treatment of HIE among the various treatment methods (6).

Mild hypothermia therapy is considered to be an important treatment that is able to improve the prognosis of HIE and greatly decrease the mortality rate and morbidity of the impairments or disabilities in infants with full-term HIE. Although the main side-effects of mild hypothermia therapy have been demonstrated to involve increased morbidity due to arrhythmia and thrombocytopenia (6), mild hypothermia has been deemed to be safe and viable as a treatment option, and its mechanism of action is nonspecific, multi-channelled, and multi-targeted. Aberrant energy metabolism in brain cells underlies HIE pathogenesis (2,3). The level and duration of aberrant energy metabolism may affect not only the short-term but also the long-term prognosis of patients. Mild hypothermia protects the brain cells through multiple mechanisms (7), whereas the effects of mild hypothermia on the brain energy metabolism of individuals with HIE remains unclear. The development of positron emission tomography/computed tomography (PET/CT), one of the most advanced medical techniques (8), provides the leading medical imaging invention worldwide. It is possible that imaging with ^18^F-fluorodeoxyglucose (^18^F-FDG) may directly reflect the state of local cellular energy metabolism. PET/CT scans have been widely applied in adults and children but are very rarely used in newborns (9).

Based on the aforementioned knowledge, the present study used ^18^F-FDG PET/CT scanning to evaluate and analyse the changes in the energy metabolism of the brain cells of patients with neonatal HIE following mild hypothermia therapy.

## Subjects and methods

### Subjects

There were 305 cases of HIE in the Neonatal Department of The Affiliated Hospital of Luzhou Medical College (Luzhou, China) from May 2012 to September 2013. Of these, 63 patients were selected for inclusion in this study.

The inclusion criteria were as follows: i) born within 6 h of labour following a ≥36-week gestation period; ii) met the diagnostic criteria for medium or severe HIE (10); iii) not treated with high-dose anti-spasmodic drugs and did not have congenital malformation, intracranial hemorrhage, scalp injury or related conditions and iv) an informed consent form was provided by the newborn’s legal guardian or family member.

In total, 37/63 patients received mild hypothermia therapy. Among them, 24 were male and 13 were female. The mean gestational age at birth was 37.55±4.21 weeks (range, 36–41±5 ±days). The mean body mass at birth was 3,390.27±573.08 g (range, 2.55–4.75 kg) and the mean age at hospital admission was 4.03±1.28 h (range, 1.7–5.7 h). The other 26 cases, 10 male and 16 female, who did not undergo mild hypothermia therapy, had a mean gestational age of 38.12±1.21 weeks (range, 36±3–40 ±5 days), mean body mass at birth of 3,393.07±473.41 g (range, 2.85–4.17 kg) and a mean age at hospital admission of 4.22±1.05 h (range 1.5–5.2 h). The general data for the two groups are summarised in Table I. In addition, 6 newborns that were hospitalized for monitoring according to their guardian’s request but were diagnosed as normal constituted a control group. The newborns were equal in gender, with a mean gestational age of 40.12±0.23 weeks (range, 39+3–40+4 weeks), a mean body mass at birth of 3250.15±335.50 g (range, 2,950–3,520 g) and a mean age at hospital admission of 4.02±0.15 h (range, 2.5–5.5 h).

This study was approved by the Ethics Committee of the Affiliated Hospital of Luzhou Medical College. Informed consent was obtained from the patients’ legal guardians.

### Grouping

Newborn patients were randomly divided into mild hypothermia therapy (n=37) and conventional therapy (n=26) groups using the coin-toss method. The conventional therapy group received a ‘three-supportive and two-symptomatic’ treatment. In this treatment: aeration and oxygenation were properly maintained to avoid hypoxemia, hyperoxia, hypercapnia or hypocapnia; brain blood perfusion was maintained to avoid severe blood pressure fluctuation; a correct blood glucose level was maintained; liquid intake was properly controlled to prevent encephaloedema and; phenobarbital was administered to prevent convulsions. In the mild hypothermia group, mild hypothermia therapy was performed in addition to conventional therapy. ^18^F-FDG PET/CT examination was performed at the time of hospital admission and seven days following treatment.

### Mild hypothermia therapy

The Olympic Cool-Cap 004204A, (Natus Medical Inc., Seattle, Washington, USA) a mild hypothermia therapeutic apparatus with a cool-cap system, was used in the current study. The cap was operated strictly according to the manual and moderate-sized cool caps were used to cover the heads of the newborns. Special temperature probes were placed at the rectum, bregma and hepatic regions, and radiation board probes for emergency treatment were placed at the brain and abdomen, according to the manufacturers’ instructions. The therapeutic apparatus was started, the temperature of the cool-cap was set and the temperatures of the different body sites were monitored. When the body temperature reached a reasonable range for mild hypothermia therapy (≤35.5°C), the settings were changed to maintain treatment. Specifically, the rectal temperature was maintained at 34–35°C and the bregma temperature at 20–25°C. If the infant’s temperature did not decrease to the expected level, the power to the radiation board was shut off, and further mild hypothermia therapy was induced. In these cases, the temperature usually reached the expected level of 35.5°C in 1–2 h. Subsequently, the radiation board was powered on and the mild hypothermia therapy was continued. Based on the dynamic information on the display screen of the therapeutic apparatus, the newborn patient’s skin was examined every 4 h and the therapy was paused at 12-h intervals to enable the operator to check the scalp and to adjust the contact site of the cool-cap as required. The therapeutic apparatus was set to begin an automatic rewarming program following the 72 h treatment period. The temperature of the radiation board was raised according to the display information until the patient’s rectal temperature increased by 0.5°C. This benchmark represented the end of rewarming and the completion of mild hypothermia therapy (11).

### Monitoring index

The glucose metabolic rates in the neonatal cerebral tissue were assessed at the time of hospitalisation and seven days following treatment using ^18^F-FDG PET/CT imaging. ^18^F-FDG was intravenously administered at a dosage of 3.7 MBq/kg and scanning was performed 40–60 min following the patient entering deep sleep. Semi-quantitative analysis was performed based on the standardised uptake values (SUVs) in the regions with abnormal glucose metabolic rates.

A radiologist with >10 years experience and a similarly senior doctor of nuclear medicine jointly read the films. A cross-section was selected and the maximum SUV value on that plane was read. The sections that crossed the site on the first plane with the maximum SUV value were then compared to obtain the overall maximum SUV value. The maximum SUV values of the brain regions, including the thalamus, basal ganglia, sensorimotor area, parietal lobe, occipital lobe and cerebellar cortex, were calculated using this method.

### PET scanner and photographic developer

A Gemini TF/T16 PET/CT scanner (Philips, Best, The Netherlands) was used to carry out the imaging. Image acquisition was performed over a 10-min interval using 3-dimensional volume scanning. CT image reconstruction was performed using filtered backprojection with a 512×512 pixel matrix and 2.5-fold magnification. ^18^F-FDG was prepared by the PET Center at The Affiliated Hospital of Luzhou Medical College The product was clear and transparent with pH 7.3, a radiochemical purity >95% and a radioactive half-life of 109 min. Bacterial cultures and pyrogen tests presented negative results.

### Statistical analysis

SPSS software, version 9.0 (SPSS, Inc., Chicago, IL, USA) was used to carry out the statistical analyses in the current study. Measurement data are presented as the mean ± standard deviation (SD); enumeration data are presented as percentages. Non-parametric and the χ^2^ tests were used to compare general information. Variance analysis and the rank-sum test were used for the analysis of differences in ^18^F-FDG PET/CT data between groups. The change in glucose metabolism rate as an effect of therapy was analysed using a paired-sample Student’s t-test. P<0.05 was considered to indicate a statistically significant difference.

## Results

### General characteristics of the study subjects

There were no differences between the two groups of patients with HIE in terms of gestational age, body mass at birth and age at hospitalisation as determined using a non-parametric test for two independent samples. There was also no difference in gender ratio, as determined by the χ^2^ test (P>0.05; Table I).

### Comparison of the glucose metabolic rates between groups

**Figure 1:**
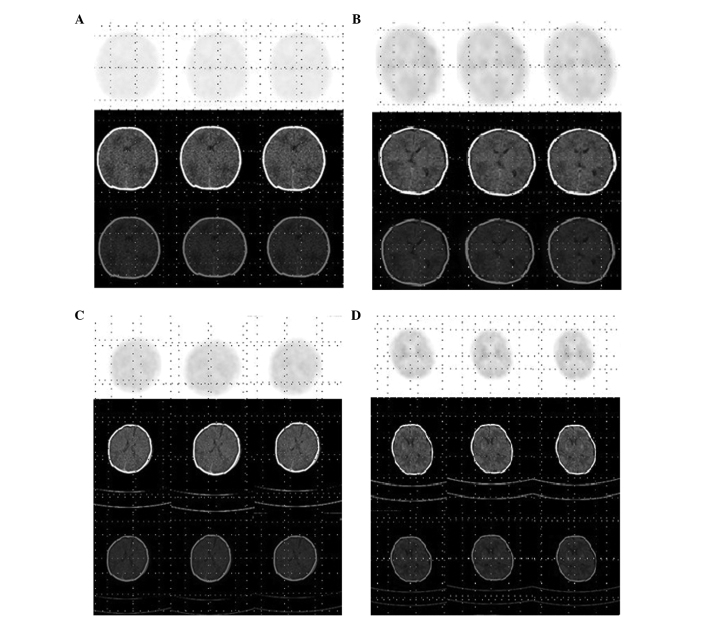
Comparisons of the rates of glucose metabolism prior to and following treatment. (A) Images prior to mild hypothermia therapy. The images show diffuse low density and low metabolism in the bilateral brain tissue. (B) Images following mild hypothermia therapy. The metabolism of the brain tissue has recovered. The brain images are sharp, clear and symmetrical. (C) Images prior to conventional therapy. The images show diffuse low density and low metabolism in the bilateral brain tissue. (D) Images following conventional therapy. The metabolism of the brain tissue has recovered significantly, but the level of recovery is lower compared with that of mild hypothermia therapy. In each figure: first row, images following positron emission tomography (PET) alone; second row, images following computed tomography (CT) and; third row, reconstructed images for PET + CT.

According to the results, the mild hypothermia and conventional therapy treatments significantly improved the rate of glucose metabolism (Table II and Fig. 1; P<0.001). There were no significant differences in the metabolic rate of each brain region, thalamus, basal ganglia, frontal lobe, parietal lobe and occipital lobe, between the two groups prior to treatment (Z=1.04, 0.13, 0.80, 1.10, 1.03; P=0.30, 0.90, 0.42, 0.28, 0.31; P>0.05). In terms of the level of improvement, mild hypothermia therapy had more promising effects on glucose metabolism rate than conventional therapy, with statistical significance (Z=4.46, 4.46, 4.46, 4.46, 4.47; P<0.0001).

## Discussion

Primary and secondary energy failure are significant constituents of the pathogenesis of neonatal HIE. Furthermore, a previous study observed a decreased rate of glucose metabolism in association with HIE (12). The present study demonstrated that the reduced rate of glucose metabolism was improved using conventional and mild hypothermia treatment approaches. Compared with the effects of conventional therapy, mild hypothermia therapy was more efficacious at improving the rate of glucose metabolism.

The pathogenesis of neonatal HIE remains unclear. Potential causes include the combined action of various factors, including primary and secondary energy exhaustion, the toxic effect of EAAs and the inflow of Ca^2+^ ions. Recently, additional factors have been discovered. Oxygen radicals, generated in brain injury-associated ischaemia reperfusion, cause cell death and further injuries to brain tissue (2,3).

Oxidative stress is a major factor in the development of reperfusion injury (13). Furthermore, inflammatory injury may also partly contribute to the occurrence of HIE. The cellular contents released as a consequence of the death and lysis of brain cells initiates the production of several pre-inflammatory and inflammatory cytokines, including interleukin (IL)-1β, IL-6, IL-8 and tumour necrosis factor (TNF)-α (2). IL-6 promotes the accumulation of neutrophils at the site of injury, which increases the permeability of endotheliocytes and aggravates cellular damage in the brain. Furthermore, TNF-α induces the degradation of the basement membrane, inhibits myelinogenesis and triggers thrombus and haemorrhage, which results in pathological lesions and cell death. Various apoptosis-promoting factors, including abnormal energy metabolism, calcium overload, excessive quantities of oxygen radicals and impairments in DNA replication may contribute to the development of ischaemia and anoxia by triggering the release of cytochrome *c*. This activates caspase 3 which initiates the apoptosis of neurons under a caspase 3-mediated pathway (3). Disorders of energy metabolism are major factors in the various forms of HIE pathogenesis.

Mild hypothermia therapy protects brain cells through various mechanisms, including the inhibition of neuronal apoptosis, which may be attributed to reduced ATP consumption, energy expenditure, the accumulation of excitatory neurotransmitters, oxygen radical generation, cytochrome *c* release and caspase 3 activation. Decreased Ca^2+^ inflow protects the brain cells and prevents the destruction of structural proteins. According to a previous study, neuronal apoptosis associated with abnormal metabolism, oxygen radicals, pro-inflammatory factors and the excessive release of EAAs usually peaks at 72 h following a stimulus (14). Since 2005, randomised controlled trials (RCTs) for systemic and selective local mild hypothermia therapy have been performed in international centres in the USA, UK, New Zealand, Canada and other countries. These have verified the safety and validity of mild hypothermia therapy for neonatal HIE (15–17).

PET/CT has become a popular procedure for the early diagnosis of various tumours. It is especially effective for distinguishing between benign and malignant states. The method has also been applied in cardiovascular and nervous system diseases, including in the diagnosis and monitoring of coronary heart conditions, the assessment of myocardium viability, the localisation of epileptogenic foci and the early differential diagnosis of Alzheimer’s and Parkinson’s diseases (18). Due to restrictions based on safety considerations, PET/CT was rarely applied in paediatrics in the past (19). In recent years, there have been numerous studies conducted on the application of PET/CT in paediatrics and neonatology (20–22). Therefore, it is reasonable to speculate that PET/CT may potentially be valuable in various other medical applications, including for the diagnosis, assessment of treatment and monitoring of prognosis in neonatal HIE (12). ^18^F-FDG is the most commonly used radioactive tracer worldwide. An isomer of glucose, it is usually absorbed and transported into the brain. The retention of FDG-6-phosphate (FDG-6-P), a metabolic end-product of ^18^F-FDG, in the brain reflects the utilisation and metabolism of glucose in brain cells (23). A local reduction in the level of blood in HIE results in the reduced utilisation of glucose and a failure to supply energy. Subsequently, histopathological changes occur in the neurons, which may account for the damage in brain tissue that occurs in the disease. PET scanning and localisation are currently used to demonstrate the extent of hypoxic damage in adult brains. However, contrary to the stable metabolic conditions observed in adults, glucose metabolism in newborns is substantially different, with physiological decreases in the frontal lobe (24) and increased glucose intake in the cortex and thalamus. The even distribution of glucose revealed in PET scans has demonstrated that glucose metabolism is stable in newborns following a meal. Given their accuracy in localisation, PET scans may be used to reveal early-stage abnormalities that are not detectable by CT scans or MRI examinations. Furthermore, the capability of PET in the assessment of neurogenesis may enhance and supplement current therapeutic schedules and early interventions.

The conventional ‘three-supportive and two-symptomatic’ therapy is beneficial for the improvement of clinical symptoms and energy metabolism. However, the present study indicated that the application of mild hypothermia therapy in addition to a conventional therapeutic strategy may achieve improved treatment results. The effect of mild hypothermia therapy on HIE was definitively identified. Although conventional therapy improved the rate of glucose metabolism, a lower-than-average cerebral glucose metabolism rate remained in the group. By contrast, the mild hypothermia therapy group revealed greater clinical effects and this difference was statistically significant. The pre-treatment data concerning the glucose metabolism rate in the cerebral regions revealed no significant differences between the conventional and mild hypothermia therapy groups. The post-treatment data indicated that the level of improvement with mild hypothermia therapy was significantly higher when compared with that of conventional therapy. In terms of safety, thrombocytopoenia occurred in one case and the patient recovered following several days without special treatment. Scalp oedema was observed in 23 cases, although these patients returned to normal in 3–5 days following the cessation of treatment. No other evident therapy-associated adverse effects were observed in the patients during the hospitalisation period.

The present study has certain limitations. Firstly, although a short-term therapeutic effect was obtained, the long-term prognosis remains to be further investigated with more information and study. It is widely accepted that the time window for mild hypothermia therapy is within 6 h of HIE onset (25,26), whereas there have been no studies, to the best of our knowledge, on the definitive effects of mild hypothermia therapy for cases in which therapy was initiated >6 h following HIE onset. Therefore, in the present study, treatment was not declined for patients who had missed the normal time window for treatment; the related materials and information were collected for further study. Furthermore, the potential adverse effects should be investigated. The procedure should be performed according to specifications to avoid related complications, including scalp oedema. The thrombocyte count, diffused intravascular coagulation indices, blood pressure and body-wide circulation should also be closely monitored.

Compared with regular CT, which only displays low-density images, the advantage of ^18^F-FDG PET/CT is that it is able to provide more information for energy metabolic analysis in brain tissue. The range of changes in glucose metabolism may help to elucidate the functional conditions of brain cells and to assess injury levels and therapeutic effects more accurately. During the examination process, the radiation capture time and the ^18^F-FDG injection should be administered under strict dosage control.

## Figures and Tables

**Table I:** Comparison of general patient information.

Group	Gestational age (weeks)	Body mass at birth (g)	Gender (male/female)	Admission age (h)
Mild hypothermia (n=37)	37.55±4.21	3390.27±573.08	24/13	4.03±1.28
Conventional (n=26)	38.12±1.21	3393.07±473.41	10/16	4.22±1.01
Statistical values	Z=0.52	Z=0.41	χ^2^=4.32	Z=0.60
P-value	0.68	0.50	0.30	0.68

Data are presented as mean±standard deviation.

**Table II:** Comparison of the rates glucose metabolic as assessed by ^18^F-fluorodeoxyglucose positron emission computed tomography (^18^F-FDG PET/CT).

Group (n)	Time point	Normal metabolic rate, n (%)	Slightly decreased metabolic rate, n (%)	Markedly decreased metabolic rate, n (%)	Increased metabolic rate, n (%)	Comparison between pre- and post-treatment
Mild hypothermia (37)	Pre-treatment	10 (27.03)	9 (24.32)	17 (45.95)	1 (2.70)	χ^2^=29.20, P<0.001
	Post-treatment	15 (40.54)	20 (54.05)	0 (0.00)	2 (5.40)	
Conventional (26)	Pre-treatment	3 (11.54)	7 (26.92)	15 (57.69)	1 (3.85)	χ^2^=8.42 P=0.003
	Post-treatment	6 (23.07)	13 (50.00)	5 (19.23)	2 (7.69)	
Control (6)	Day 1	6 (100.0)	0 (0.00)	0 (0.00)	0 (0.00)	-
	Day 7	0 (0.00)	0 (0.00)	0 (0.00)	0 (0.00)	-
